# Deficiency in steroid receptor coactivator 3 enhances cytokine production in IgE-stimulated mast cells and passive systemic anaphylaxis in mice

**DOI:** 10.1186/2045-3701-4-21

**Published:** 2014-04-23

**Authors:** Xiaochun Xia, Wei Wan, Qiang Chen, Kun Liu, Sidra Majaz, Pingli Mo, Jianming Xu, Chundong Yu

**Affiliations:** 1 State Key Laboratory of Cellular Stress Biology, Innovation Center for Cell Biology, School of Life Sciences, Xiamen University, Xiang-An South Road, Xiamen, Fujian 360112, China; 2 The First Affiliated Hospital of Xiamen University, Xiamen, China; 3 Department of Molecular and Cellular Biology, Baylor College of Medicine, Houston, TX, USA

**Keywords:** SRC-3, Mast cell, Passive systemic anaphylaxis, Passive cutaneous anaphylaxis

## Abstract

**Background:**

Steroid receptor coactivator 3 (SRC-3) is a multifunctional protein that plays an important role in malignancy of several cancers and in regulation of bacterial LPS-induced inflammation. However, the involvement of SRC-3 in allergic response remains unclear. Herein we used passive systemic anaphylaxis (PSA) and passive cutaneous anaphylaxis (PCA) mouse models to assess the role of SRC-3 in allergic response.

**Results:**

SRC-3-deficient mice exhibited more severe allergic response as demonstrated by a significant drop in body temperature and a delayed recovery period compared to wild-type mice in PSA mouse model, whereas no significant difference was observed between two kinds of mice in PCA mouse models. Mast cells play a pivotal role in IgE-mediated allergic response. Antigen-induced aggregation of IgE receptor (FcϵRI) on the surface of mast cell activates a cascade of signaling events leading to the degranulation and cytokine production in mast cells. SRC-3-deficient bone marrow derived mast cells (BMMCs) developed normally but secreted more proinflammatory cytokines such as TNF-α and IL-6 than wild-type cells after antigen stimulation, whereas there was no significant difference in degranulation between two kinds of mast cells. Further studies showed that SRC-3 inhibited the activation of nuclear factor NF-κB pathway and MAPKs including extracellular signal-regulated kinase (ERK), c-jun N-terminal kinase (JNK), and p38 in antigen-stimulated mast cells.

**Conclusions:**

Our data demonstrate that SRC-3 suppresses cytokine production in antigen-stimulated mast cells as well as PSA in mice at least in part through inhibiting NF-κB and MAPK signaling pathways. Therefore, SRC-3 plays a protective role in PSA and it may become a drug target for anaphylactic diseases.

## Background

Mast cells play a pivotal role in IgE-dependent allergic diseases such as allergic rhinitis, asthma and anaphylaxis [1,2]. IgE antibodies and mast cells have been convincingly linked to the pathology of anaphylaxis [3]. Aggregation of the high affinity IgE receptor (FcϵRI) on the surface of mast cell activates a cascade of signaling events leading to the degranulation and cytokine synthesis in mast cells. Mast cell exerts its effect on various IgE-dependent or IgE-independent immune responses not only through the release of degranules and cytokines but also through cell-cell interaction [4]. Moreover mast cell progenitors in the bone marrow can be induced by interleukin-3 (IL-3) to further proliferate and differentiate into bone marrow-derived mast cells [4,5].

Steroid receptor coactivator-3 (SRC-3/AIB1/ACTR/pCIP/RAC3/TRAM-1) is a member of p160 coactivator family that also includes SRC-1 and SRC-2, which interacts with nuclear receptors and other transcription factors to enhance their effects on target gene transcription [6-9]. SRC-3 deficiency results in growth retardation and decrease of reproduction rate [10]. Besides that, it also plays an important role in many physiological and pathologic events such as cell growth, oncogenesis and differentiation [11-14]. Studies showed that SRC-3 is overexpressed in many tumors [13,15], while other studies displayed SRC-3 functions as a tumor suppressor [14,16]. Therefore, the oncogenic or tumor suppressor effect of SRC-3 depends on the cell context. We have previously demonstrated that SRC-3^-/-^ mice are highly susceptible to LPS-induced lethality [17] and are markedly susceptible to the lethality caused by *E. coli-*induced peritonitis [18]. In addition, SRC-3 represses the production of proinflammatory cytokines including TNF-α, IL-1β and IL-6 through inhibiting cytokine mRNA translation [17]. These results indicate that SRC-3 can suppress inflammatory response. However, the function of SRC-3 in allergic response and inflammation remains unknown.

Anaphylaxis is a severe, systemic allergic reaction involving the respiratory and cardiovascular systems, usually with additional cutaneous and/or gastrointestinal features [19]. Traditional treatments for allergic diseases have some limitations such as efficacy deficiency or severe side effect, thus new targets are being explored for development of new drugs. In this study, we used SRC-3^-/-^ mice to determine the role of SRC-3 in IgE-mediated anaphylaxis. We found that SRC-3^-/-^ mice suffered severe passive systemic anaphylaxis than wild-type mice. In addition, SRC-3 suppressed cytokine production in antigen-stimulated mast cells at least in part through inhibiting MAPK and NF-κB pathways. These results demonstrate that SRC-3 plays a protective role in passive systemic anaphylaxis.

## Results

### Enhanced passive systemic anaphylaxis in SRC-3^-/-^mice

**Figure 1:**
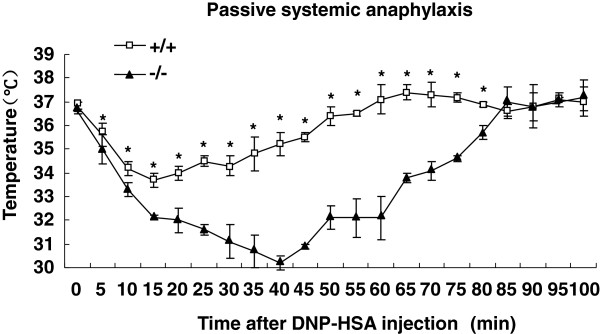
**Passive systemic anaphylaxis in wild-type and SRC-3**^**-/- **^**mice.** SRC-3^+/+^ (n = 5) and SRC-3^-/-^ mice (n = 5) were sensitized with anti-DNP IgE and DNP-HSA to induced systemic anaphylaxis as described in methods. Passive systemic anaphylaxis was monitored by measuring rectal temperatures after DNP-HSA challenge. Data represent the mean rectal temperature ± SD. *p<0.05 versus SRC-3^+/+^ mice by t-test.

To determine the *in vivo* role of SRC-3 in allergy, we examined the mast cell dependent, IgE-mediated PSA reaction, an extreme form of allergic response [20], in SRC-3^-/-^ and wild-type mice. Passive systemic anaphylaxis was elicited by injecting of 10 μg anti-DNP IgE intravenously, 24 hrs later, mice were administrated with DNP-human serum albumin (DNP-HSA) antigen by intravenously injection, and then core body temperature was monitored at indicated time intervals. As shown in Figure 1, the body temperature of mice dropped after DNP-HSA injection, and a greater drop was observed in SRC-3^-/-^ mice compared to wild-type mice. The recovery of body temperature began at 15 min in wild-type mice while this event occurred at 40 min in SRC-3^-/-^ mice. These results suggest that the allergic reaction is more severe in SRC-3^-/-^ mice compared to wild-type mice in PSA animal model.

### No significant difference in passive cutaneous anaphylaxis between SRC-3^-/-^ and wild-type mice

**Figure 2:**
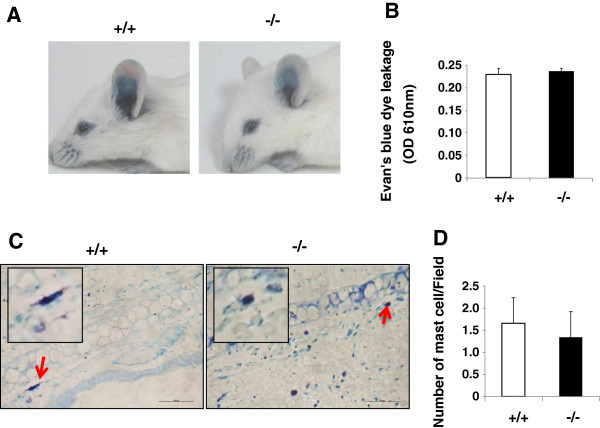
**Passive cutaneous anaphylaxis in SRC-3**^**+/+ **^**and SRC-3**^**-/- **^**mice.** SRC-3^+/+^ (n = 6) and SRC-3^-/-^ mice (n = 6) were sensitized with anti-DNP IgE and DNP-HSA to induce cutaneous anaphylaxis as described in methods **(A-D)**. **A**, dye extravasation was observed after DNP-HSA injection at the injection sites in the ears. Photographs of the mice were taken 90 min after DNP-HSA administration. Representative images are shown. **B**, Extravasation of Evan’s blue was quantified as described in methods. Values are expressed as means + SD from three independent experiments. **C**, Toludine blue staining of mast cells in the ear skin of SRC-3^+/+^ and SRC-3^-/-^ mice after antigen challenge. Representative images are shown; arrows indicate degranulated tissue mast cells. **D**, mast cells were quantified, values are expressed as means + SD from three independent experiments.

To further investigate the role of SRC-3 in anaphylaxis, we performed another allergic mouse model named passive cutaneous anaphylaxis (PCA). In PCA, local extravasation is induced by local injection of anti-DNP IgE and intravenous injection of DNP-HSA [21]. The ears of both wild-type and SRC-3^-/-^ mice were intradermally injected with anti-DNP IgE, then DNP-HSA and Evan’s blue dye were injected 24 h later. After IgE and DNP-HSA treatment, the vascular permeability increased to allow the Evan’s blue dye to leak from the blood vessels. As shown in Figure 2A-D, Evan’s blue dye leakage was observed in both SRC-3^-/-^ and wild-type mice. However, there was no significant difference in the extent of dye leakage between these two kinds of mice.

### No significant difference in maturation and antigen-stimulated degranulation between SRC-3^-/-^ and wild-type BMMCs

**Figure 3:**
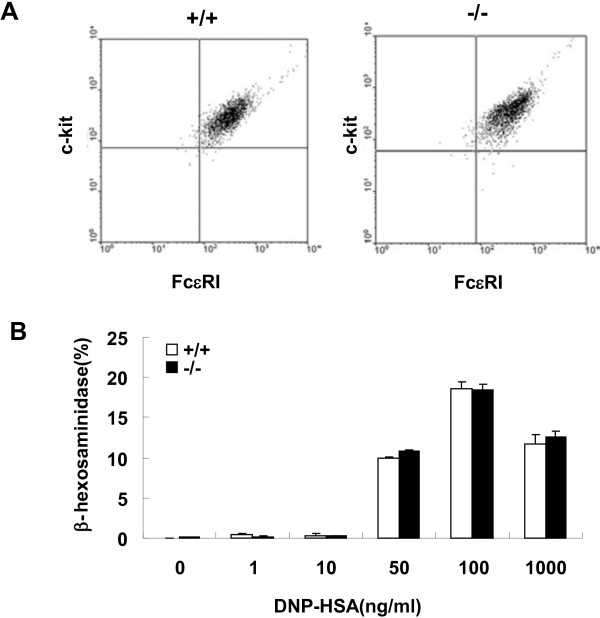
**The degranulation of SRC-3**^**+/+ **^**and SRC-3**^**-/- **^**BMMCs. (A)** Identification of BMMCs. Bone marrow cells were obtained from BALB/c mice and cultured in BMMC-complete medium. After 5 weeks, cells were identified by flow cytometric analysis for FcϵRI and c-kit expression. The experiment was repeated for 3 ~ 5 times. Representative results are shown from three independent experiments. **(B)** BMMCs were stimulated with IgE and different concentration of DNP-HSA. Degranulation was measured by assessing hexosaminidase activity in the media or cell lysates. Values are shown as the mean + SD from three independent experiments.

To further assess the function of SRC-3 in mast cell-mediated anaphylaxis, BMMCs were used. Mast cell progenitors in the bone marrow can be induced by IL-3 to further proliferate and differentiate into BMMCs. Mature BMMCs express several kinds of receptors, among which FcϵRI and c-kit are most well-known [22]. Therefore, BMMCs were identified by flow cytometric analysis for FcϵRI and c-kit expression after incubation of SRC-3^-/-^ and wild-type bone marrow cells with BMMC complete medium for 5 weeks. As shown in Figure 3A, more than 98% cells expressed FcϵRI and c-kit, but there was no significant difference between SRC-3^-/-^ and wild-type BMMCs, indicating that SRC-3 deficiency does not affect the development and maturation of BMMCs.

It has been demonstrated that IgE-mediated mast cell activation and allergic response show the features of degranulation and inflammatory mediator production [23]. To determine the impact of SRC-3 deficiency on antigen-stimulated mast cell degranulation, we measured the levels of β-hexosaminidase, which is frequently used as a degranulation marker released from DNP-HSA-stimulated BMMCs. As shown in Figure 3B, mast cells released β-hexosaminidase after DNP-HSA treatment. However, the degranulation of antigen-stimulated BMMCs from SRC-3^-/-^ and wild-type mice showed no significant difference. This data suggest that enhanced anaphylaxis in SRC-3^-/-^ mice are not due to enhanced degranulation.

### Increased antigen-stimulated expression of IL-6 and TNF-α in SRC-3^-/-^ BMMCs

**Figure 4:**
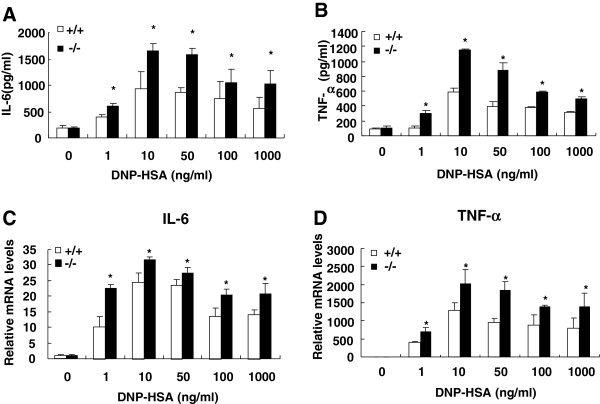
**Increased antigen-stimulated IL-6 and TNF-α expression from SRC-3**^**-/- **^**BMMCs compared with SRC-3**^**+/+ **^**BMMCs.** BMMCs were pretreated with 1 μg/ml anti-DNP IgE for 4 h and then were stimulated with different concentration of DNP-HSA overnight at 37°C in 5% CO_2_. **(A and B)** The amounts of IL-6 and TNF-α in the medium were measured using ELISA assay kits. **C** and **D**, The mRNA levels of IL-6 and TNF-α were measured by real-time PCR. Values are shown as the mean + SD from three independent experiments. *p < 0.05.

Upon stimulation, mast cells synthesize and secrete proinflammatory cytokines [21,24]. Cytokines such as IL-6 and TNF-α are important for allergic inflammation mediated by mast cell activation. To investigate whether SRC-3 could regulate cytokine production, IL-6 and TNF-α protein levels were measured in the cell supernatants of antigen-stimulated BMMCs. As shown in Figure 4A and B, SRC-3^-/-^ BMMCs produced more IL-6 and TNF-α than wild-type BMMCs after different concentrations of antigen treatment overnight (p < 0.05). Further study showed that the mRNA levels of IL-6 and TNF-α in SRC-3^-/-^ BMMCs were higher than that in wild-type BMMCs after antigen stimulation (p < 0.05). These results indicate that SRC-3 inhibits cytokine production at both protein and mRNA levels.

### Activation of Syk and PLCγ1 in antigen-stimualted BMMCs is independent of SRC-3

**Figure 5:**
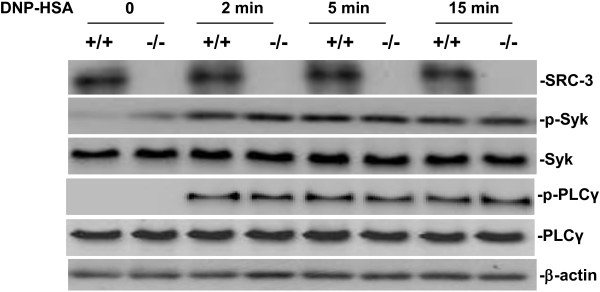
**SRC-3 deficiency has no effect on activation of Syk and PLCγ.** BMMCs were stimulated with anti-DNP IgE 1 μg/ml for 4 h, and then treated with DNP-HSA 10 ng/ml for various times. Whole cell lysates were analyzed by western-blotting for Syk, p-Syk, PLCγ and p-PLCγ. Representative images are shown from three independent experiments.

In mast cell, re-exposure to allergen triggers cross-linking of IgE/FcϵRI, which activates Src family protein tyrosine kinases such as Lyn and Fyn. Lyn initially phosphorylates Syk and then activates phospholipase (PL)Cγ1, a critical enzyme for generation of the calcium signal for degranulation [25]. We investigated the activation of Syk and PLCγ1 in antigen-stimulated BMMCs. As shown in Figure 5, phosphorylation of Syk and PLCγ1 was induced in antigen-stimulated BMMCs, but there was no difference between SRC-3^-/-^ and wild-type BMMCs. These results are consistent with the observation that there was no difference in degranulation between SRC-3^-/-^ and wild-type mice.

### Increased activation of IκB kinase (IKK)-IκB-NF-κB pathway in antigen-induced SRC-3^-/-^ BMMCs

**Figure 6:**
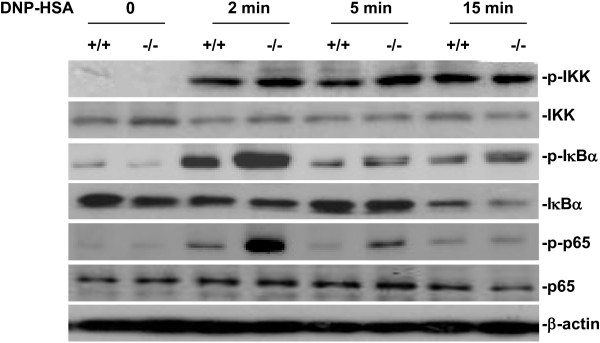
**SRC-3 deficiency leads to increased activation of IKK-IκB-NF-κB pathway.** BMMCs were stimulated with anti-DNP IgE 1 μg/ml for 4 h, and then treated with DNP-HSA 10 ng/ml for various times. Whole cell lysates were analyzed by western-blotting for IKK, p-IKK, IκB, p-IκB, p65 and p-p65. Representative images are shown from three independent experiments.

The cross-linking of allergen-specific IgE bound to its high-affinity receptor FcϵRI results in a series of molecular events leading to NF-κB activation and subsequent cytokine production [26,27]. Phosphorylation and degradation of IκB allow NF-κB to translocate to nucleus and to bind to DNA to initiate gene expression. IKK is responsible for IκB phosphorylation and plays a critical role in the initiation of IKK-IκB-NF-κB cascade following FcϵRI crossing linking in mast cells [28]. Since SRC-3^-/-^ BMMCs produced more IL-6 and TNF-α compared with wild-type BMMCs after IgE and antigen stimulation, we explored whether SRC-3 exerts its effect on IKK-IκB-NF-κB pathway. As shown in Figure 6, although the phosphorylation of IKK was unaltered, the phosphorylation of IκB and NF-κB subunit p65 were enhanced and the degradation of IκB was increased in SRC-3^-/-^ BMMCs. These data demonstrate enhanced activation of IKK-IκB-NF-κB pathway in SRC-3^-/-^ BMMCs.

### Increased activation of MAPKs in antigen-induced SRC-3^-/-^ BMMCs

**Figure 7:**
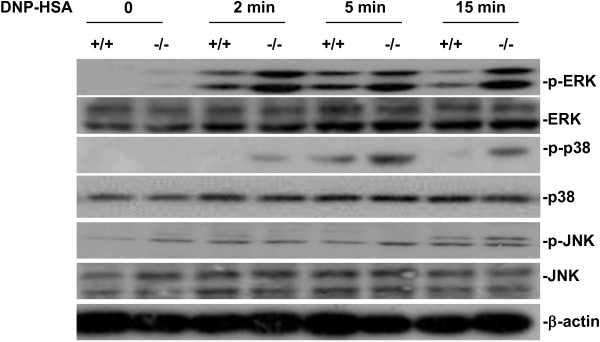
**SRC-3 deficiency leads to increased MAPK pathway activation.** BMMCs were stimulated with anti-DNP IgE 1 μg/ml for 4 h, and then treated with DNP-HSA 10 ng/ml for various times. Whole cell lysates were analyzed by western-blotting for ERK, p-ERK, JNK, p-JNK, p38 and p-p38. Representative images are shown from three independent experiments.

Given that SRC-3^-/-^ BMMCs produced more cytokines including IL-6 and TNF-α than wild-type BMMCs, and activation of MAPKs such as ERK1/2, p38, and JNK are also required for cytokine production by BMMCs in response to antigen stimulation [25], We examined MAPK signaling in antigen-induced SRC-3^-/-^ and wild-type BMMCs. As shown in Figure 7, the phosphorylation of ERK1/2, p38, and JNK was increased more in antigen-stimulated SRC-3^-/-^ BMMCs than that in wild-type BMMCs. These results indicate that MAPK activation is enhanced in SRC-3^-/-^ BMMCs.

## Discussion

In this study, we found that SRC-3^-/-^ mice suffered more severe IgE-induced passive systemic anaphylaxis but not passive cutaneous anaphylaxis compared to wild-type mice, indicating a potential role of SRC-3 in anaphylaxis. Mast cells were considered to be the important effector cells for IgE-induced anaphylaxis. The high affinity receptor for IgE (FcϵRI) is critical for mast cell development and function [29]. Cross-linking of FcϵRI by antigen and IgE results in mast cell degranulation and cytokine production [21]. Given that both mast cell degranulation and cytokine production contribute to the severity of IgE-induced passive systemic anaphylaxis, whereas the severity of passive cutaneous anaphylaxis is mainly affected by mast cell degranulation, we hypothesized that SRC-3 may affect cytokine production but not degranulation by mast cells since SRC-3 deficiency only affected IgE-induced passive systemic anaphylaxis but not passive cutaneous anaphylaxis.

To corroborate the role of SRC-3 in IgE-dependent mast cell activation, we obtained mouse BMMCs from SRC-3^-/-^ and wild-type mice and treated them with IgE/DNP-HSA. Degranulation and cytokine production were observed in both SRC-3^-/-^ and wild-type BMMCs after stimulating with IgE/DNP-HSA. However, SRC-3^-/-^ BMMCs released higher amounts of IL-6 and TNF-α than that from wild-type BMMCs. Therefore, more severe passive systemic anaphylaxis in SRC-3^-/-^ mice is due to increased cytokine production in mast cells. No difference was observed in degranulation between wild-type and SRC-3^-/-^ BMMCs, explaining no significant difference in passive cutaneous anaphylaxis between wild-type and SRC-3^-/-^ mice.

In antigen-stimulated mast cells, Src family kinases such as Lyn and Fyn are activated and subsequently phosphorylate Syk, a central signaling molecule that can activate the downstream signaling molecules including PLCγ1 and MAPKs [30]. Activation of PLCγ1 can increase Ca2^+^ influx, which is a key regulator for mast cell degranulation [31]. Our study showed no difference in the activation of Syk and PLCγ1 in antigen-stimulated mast cells, explaining that there was no difference in degranulation between SRC-3^-/-^ and wild-type BMMCs. Activation of NF-κB and MAPKs including ERK1/2, JNK and p38 is important for the cytokine production in mast cells [32]. Cross-linking of allergen-specific IgE bound to its receptor FcϵRI induced a series of molecular events leading to NF-κB and MAPK activation and subsequent cytokine production [26,27]. Since NF-κB and MAPKs including ERK1/2, JNK and p38 are important for the cytokine production in mast cells [32], and SRC-3^-/-^ BMMCs produced more cytokines than wild-type BMMCs, we examined whether SRC-3 exerts its effect on NF-κB and MAPK signaling pathways. Our results showed that the phosphorylation of IκB and NF-κB subunit p65, ERK1/2, JNK and p38 were increased in antigen-stimulated SRC-3^-/-^ BMMCs as compared to wild-type BMMCs, suggesting that SRC-3 negatively regulates NF-κB and MAPK signaling pathways.

IKK is known to be responsible for the phosphorylation of IκB and p65 and the subsequent activation of NF-κB. However, the phosphorylation of IKK was not affected by SRC-3 deficiency, indicating that SRC-3 inhibits the activation of NF-κB downstream of IKK. It has been shown that SRC-3 is able to associate with IKKs to enhance or suppress the activation of NF-κB on cellular context dependent manner [14,16]. In our study, we also found that SRC-3 and IKKβ had a physical interaction (Additional file 1: Figure S1A). Among five functional domains of SRC-3 which includes basic-helix-loop-helix (bHLH) domain, serine/threonine (S/T) domain, receptor interaction domain (RID), CBP/P300 interaction domain (CID), and histone acetyltransferase (HAT) domain, S/T and HAT domains of SRC-3 interacted with IKKβ (Additional file 1: Figure S1B). Therefore, SRC-3 may interact with IKKβ through these two domains to negatively regulate IKKβ activity in mast cells in response to antigen stimulation.

It is interesting that SRC-3 could inhibit IgE-induced activation of MAPK pathway by blocking the phosphorylation of JNK, p38 and ERK. JNK, p38 and ERK have been shown to be able to phosphorylate multiple sites on SRC-3 [33], implicating that JNK, p38 and ERK has the interaction with SRC-3. Therefore, it is possible that the interaction of SRC-3 with JNK, p38 and ERK may negatively affect the phosphorylation of these MAPKs by their upstream kinases. Further studies are needed to examine this possibility.

## Materials and methods

### Materials

RPMI 1640, DMSO, sodium pyruvate, antibody against β-actin and flag, anti-DNP IgE (clone SPE-7) were obtained from Sigma Aldrich (Sigma, St Louis, MO, USA); DNP-human serum albumin (DNP-HSA) was purchased from Biosearch Technologies (Biosearch Technologies, Novato CA, USA); recombinant murine SCF and IL-3 were purchased from peprotech (Peprotech, Rocky Hill, NJ, USA); anti-mouse CD117 (c-Kit)-PE, anti-mouse FcϵRI-FITC, IL-6 ELISA kit, and TNF-α ELISA kit were obtained from eBioscence (eBioscence, San Diego, CA, USA); nonessential amino acid was purchased from Gibco (Gibco, Grand Island, NY, USA); 2-mercaptoethanol was obtained from AMRESCO (AMRESCO, solon OH, USA); fetal bovine serum (FBS) was obtained from Hyclone (Thermo scientific, IL, USA); antibodies against IKKβ, phospho-p38, p38, phospho-JNK, JNK, phospho-ERK1/2, ERK1/2, p65, phospho-p65, PLCγ1, phospho-PLCγ1 and SRC-3 were purchased from Cell Signaling Technology (Danvers, MA, USA); antibody against IκBα and Syk was obtained from Santa Cruz Biotechnology (Santa Cruz, CA, USA); antibody against phospho-Syk was purchased from ProSci (ProSci INCORPORATED, CA, USA).

Female SRC-3^-/-^ mice and wild-type littermates (6–8 weeks age) on a BALB/c background were used for all experiments. Animals were maintained with specific pathogen free air at a temperature between 20 and 23°C with 12-h light and dark cycles and relative humidity of 50%. Animal experiments were performed in accordance with the Guide for the Care and Use of Laboratory Animals. All animal experimental procedures were approved by Animal Care and Use Committee of Xiamen University (Protocol Number: XMULAC20120001). Every effort was made to reduce the suffering of animals.

## Methods

### Preparation of bone marrow mast cells (BMMCs)

BMMCs were cultured as described with modifications [4,5]. Bone marrow cells were obtained from mice and cultured in BMMC-complete medium comprising RPMI 1640, 10% FBS, 100 U/ml penicillin, 100 μg/ml streptomycin, 100 mM nonessential amino acids, 1 mM sodium pyruvate, 50 μM 2-mercaptoethanol, 10 ng/ml mouse IL-3, and 10 ng/ml mouse SCF. Nonadherent cells were transferred to fresh complete medium once a week. After 4–5 weeks, cell purity was determined by flow cytometric analysis for FcϵRI and c-kit expression.

### β-Hexosaminidase (β-HEX) release assay

β-HEX release assay was performed as previously described with modification [5,34,35]. BMMCs were deprived of SCF overnight, and then sensitized in complete RPMI 1640 with 1 μg/ml anti-DNP IgE for 4 h. Cells were washed once in Tyrode’s buffer (130 mM NaCl, 10 mM HEPES, 1 mM MgCl_2_, 5 mM KCl, 1.4 mM CaCl_2_, 5.6 mM glucose, and 1 mg/ml BSA, pH 7.4) and resuspended in Tyrode’s buffer at 2 × 10^5^ per well in 96 wells. Then, cells were stimulated with DNP-HSA for 1 h. After induction, cells were harvested with 1% Triton X-100, and cell lysis or medium were mixed with *p*-nitrophenyl-*N*-acetyl-β-D-glucosamide (1 mM). The reaction was terminated by 100 μl of 0.2 M glycine (pH 10.7) after incubating for 1 h, and OD was read at a wavelength of 405 nm. Values are expressed as the percentage of intracellular *β*-hexosaminidase released into the medium.

### Cytokines assay

Measurement of IL-6 and TNF-α production by BMMCs was performed as described with modifications [35]. BMMCs were deprived of SCF overnight, and then sensitized in complete RPMI 1640 with 1 μg/ml anti-DNP IgE for 4 h. Cells were stimulated by different concentration of DNP-HSA overnight. The levels of IL-6 and TNF-α in medium were measured using ELISA kits according to the manufacturer’s instructions.

### Passive systemic anaphylaxis (PSA)

PSA was performed as previously described with modification [4,21]. Mice were injected intravenously with 10 μg anti-DNP IgE in 200 μl PBS via tail vein. 24 h later, 1 mg DNP-HSA in 200 μl PBS was injected intravenously. After DNP-HSA challenge, body temperature was monitored at various intervals using a rectal digital thermometer. Investigators were blinded to genotype during all experiments.

### Passive cutaneous anaphylaxis (PCA)

PCA was performed as previously described with modification [21]. Mice were lightly anesthetized, the right ears were injected intradermally with 1 μg anti-DNP IgE in 20 μl PBS, and the left ears were injected with 20 μl PBS as control. 24 hrs later, mice were injected intravenously with 200 μl of 1% Evan’s blue dye containing 100 μg DNP-HSA. The mice were killed after injection with DNP-HSA, and the ears were removed and incubated in 1 ml formamide at 54°C for 48 h. Absorbance of the resulting supernatants was measured at 610 nm. The relationship between Evans blue concentration and absorbance was linear, indicating that the absorbance represented the quantity of Evans blue extravasation.

### Quantitative real-time PCR

Total RNA was isolated with Trizol reagent (Invitrogen) according to the manufacturer’s instructions. The cDNA was synthesized from 2 μg of total RNA using MMLV transcriptase (ToYoBo, Shanghai, China) with random primers, Real-time PCRs were performed using SYBR Premix ExTaq (TaKaRa, Dalian, China). Quantification was normalized to the amount of endogenous GAPDH. Primers used for Real-time PCR are listed on Table 1.

**Table 1:** Real-time PCR primers

**Gene**	**Forward primers**	**Reverse primers**
mIL-6	AACGATGATGCACTTGCAGA	CTCTGAAGGACTCTGGCTTTG
mTNF-α	ACGTGGAACTGGCAGAAGAG	GGTCTGGGCCATAGAACTGA
GAPDH	GACCACAGTCCATGCCATCAC	CATACCAGGAAATGAGCTTGAC

### Western blot analysis

Cells were lysed with lysis buffer (200 mM Tris–HCl (pH 7.5), 1.5 M NaCl, 10 mM EDTA, 25 mM sodium pyrophosphate,10 mM glycerolphosphate, 10 mM sodium orythovanadate, 50 mM NaF, 1 mM PMSF, in combination with protein inhibitor cocktail). Thirty micrograms of protein lysates of each sample was subjected to SDS-PAGE and transferred onto nitrocellulose membranes. Blots were incubated with the specific primary antibodies overnight at 4°C. After being washed three times for 15 min each with TBST (TBS + 0.1% Tween20), blots were incubated with horseradishperoxidase-conjugated secondary antibody (Pierce, Rockford, IL, USA) and visualized by chemiluminescence. The band density was quantified by densitometry using Scion Image software and normalized to β-actin levels.

### Cell transfection

Cells were transfected with the indicated plasmids by using Calcium chloride. At 48 hours post-transfection, cells were harvested and then used for further experiments.

### Coimmunoprecipitation assay

For coimmunoprecipitation (Co-IP) assay, cells were lysed with lysis buffer. Cell lysates were immunoprecipitated by correspondent antibodies or control immunoglobulin G (IgG). After extensive washing, precipitates were analyzed by Western blotting.

### Statistical analysis

Data were collected from several independent experiments, with three replicates per experiment. All data were analyzed with one-way ANOVA with post-Tukey’s post test in SPSS 11.0 and p < 0.05 was considered statistically significant. Bars in the graph represent standard deviation (S.D.).

## Competing interests

The authors declare that they have no competing interests.

## Authors’ contributions

XX and CY designed the overall study; XX, WW, PM, QC, KL, MS performed the experiments and data analysis; JX provided animals; XX and CY wrote the manuscript. All authors read and approved the final manuscript.

## Supplementary Material

Additional file 1: Figure S1 IKKβ interacts with SRC-3 through the S/T and HAT domains of SRC-3. **(A)** Co-IP analysis of the interaction between SRC-3 protein and IKKβ protein in 293 T cells. **(B)** SRC-3 interacted with IKKβ through its S/T and HAT domains. Click here for file
