# Supplementary material for: Deficiency in steroid receptor coactivator 3 enhances cytokine production in IgE-stimulated mast cells and passive systemic anaphylaxis in mice
Source: Cell Biosci. 2014 Apr 23;4:21. doi: 10.1186/2045-3701-4-21 (PMC4021842; doi:10.1186/2045-3701-4-21)
Supplement: Additional file 1: Figure S1 — IKKβ interacts with SRC-3 through the S/T and HAT domains of SRC-3. (A) Co-IP analysis of the interaction between SRC-3 protein and IKKβ protein in 293 T cells. (B) SRC-3 interacted with IKKβ through its S/T and HAT domains. [file 2045-3701-4-21-S1.ppt]

## Slide 1
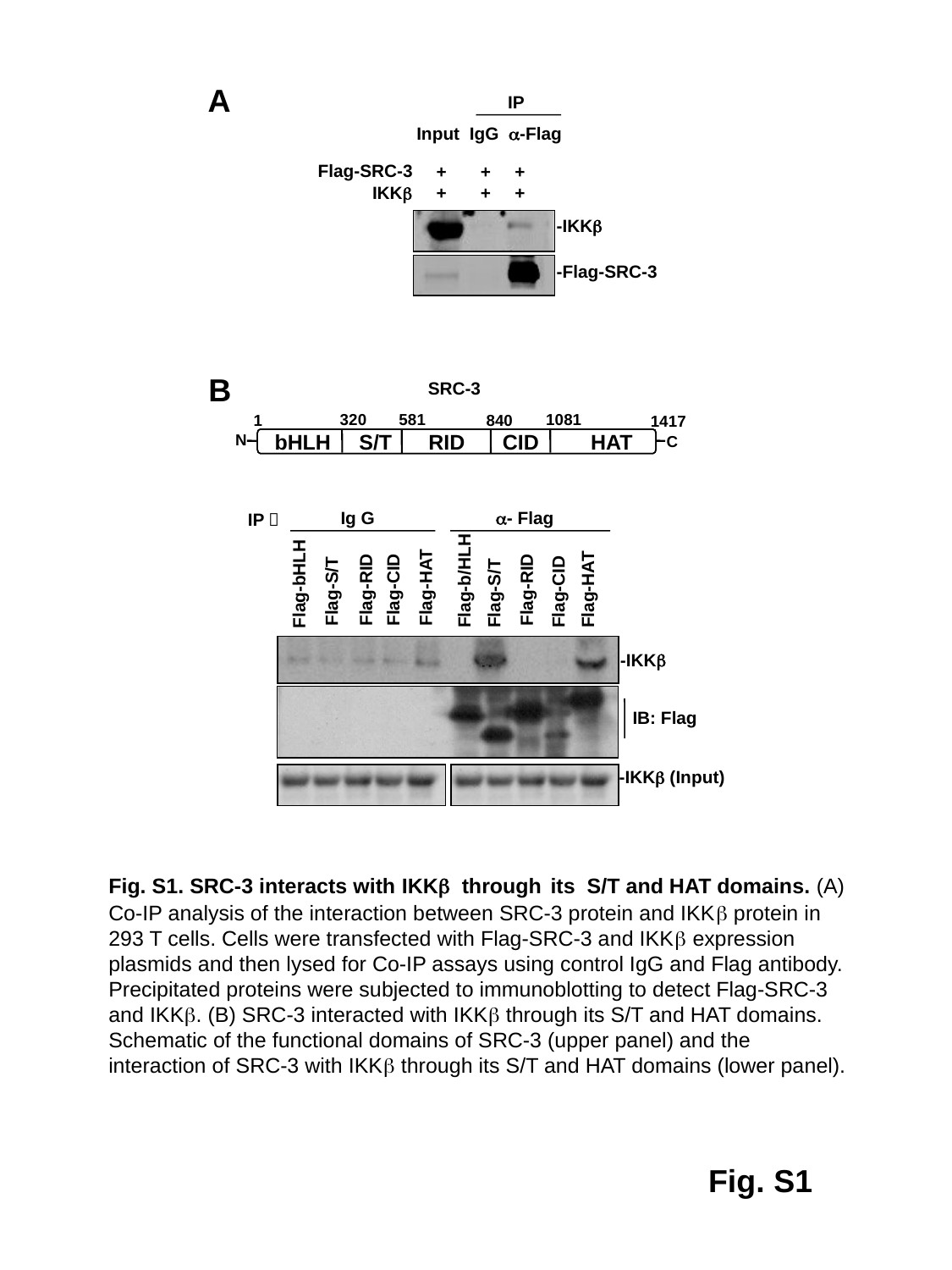

A
IP
Input IgG α-Flag
Flag-SRC-3
+ + +
+ + +
IKKβ
-IKKβ
-Flag-SRC-3
B
SRC-3
320
581
1081
1
840
1417
bHLH
S/T
RID
CID
HAT
N
S/T
C
Flag-S/T
Flag-RID
Flag-CID
Flag-HAT
Flag-RID
Flag-b/HLH
Flag-S/T
Flag-CID
Flag-HAT
Flag-bHLH
Ig G
α- Flag
IP：
-IKKβ
IB: Flag
-IKKβ (Input)
Fig. S1. SRC-3 interacts with IKKβ through its S/T and HAT domains. (A) Co-IP analysis of the interaction between SRC-3 protein and IKKβ protein in 293 T cells. Cells were transfected with Flag-SRC-3 and IKKβ expression plasmids and then lysed for Co-IP assays using control IgG and Flag antibody. Precipitated proteins were subjected to immunoblotting to detect Flag-SRC-3 and IKKβ. (B) SRC-3 interacted with IKKβ through its S/T and HAT domains. Schematic of the functional domains of SRC-3 (upper panel) and the interaction of SRC-3 with IKKβ through its S/T and HAT domains (lower panel).
Fig. S1
